# Hydrophilic Silver Nanoparticles Loaded into Niosomes: Physical–Chemical Characterization in View of Biological Applications

**DOI:** 10.3390/nano9081177

**Published:** 2019-08-17

**Authors:** Federica Rinaldi, Elena del Favero, Johannes Moeller, Patrizia Nadia Hanieh, Daniele Passeri, Marco Rossi, Livia Angeloni, Iole Venditti, Carlotta Marianecci, Maria Carafa, Ilaria Fratoddi

**Affiliations:** 1 Center for Life Nano Science@Sapienza, Istituto Italiano di Tecnologia (ITT), 00161 Rome, Italy; 2 Department of Medical Biotechnology and Translational Medicine, University of Milan, 20122 Milano, Italy; 3 ESRF Grenoble Fr, 38043 Grenoble, France; 4 Department of Drug Chemistry and Technologies, Sapienza University, 00185 Rome, Italy; 5 Department of Basic and Applied Sciences for Engineering, Sapienza University, 00185 Rome, Italy; 6 Department of Sciences, Roma Tre University, 00146 Rome, Italy; 7 Department of Chemistry, Sapienza University, 00185 Rome, Italy

**Keywords:** niosomes, silver nanoparticles, liposomes, plasmonic materials, drug delivery, nanocarriers

## Abstract

Silver nanoparticles (AgNPs) are widely used as antibacterial agents and anticancer drugs, but often their low stability limits their mass production and broad applications. The use of niosomes as a carrier to protect and envelop AgNPs gives a new perspective to solve these problems. In this study, AgNPs were functionalized with sodium 3-mercapto-1-propanesulfonate (3MPS) to induce hydrophilic behavior, improving loading in Tween 20 and Span 20 niosomes (NioTw20 and NioSp20, respectively). Entrapment efficiency was evaluated by UV analyses and is around 1–4%. Dimensions were investigated by means of dynamic light scattering (DLS) (<2R_H_> = 140 ± 4 nm and <2R_H_> = 251 ± 1 nm respectively for NioTw20 + AgNPs and NioSp20 + AgNPs) and were compared with those by atomic force microscopy (AFM) and small angle X ray scattering (SAXS) analyses. Stability was assessed in water up to 90 days, and both in bovine serum and human serum for up to 8 h. In order to characterize the local structure of niosomes, SAXS measurements have been performed on Tween 20 and Span 20 empty niosomes and loaded with AgNPs. The release profiles of hydrophilic probe calcein and lipophilic probe Nile Red were performed in HEPES buffer and in human serum. All these features contribute to conclude that the two systems, NioTw20 + AgNPs and NioSp20 + AgNPs, are suitable and promising in the field of biological applications.

## 1. Introduction

It is well known that the body barrier to external pathogenic attacks is represented by the skin, which prevents microbial invasion, so every damage or wound can provide an environment for microbial growth, leading to infection and prolonged wound healing [1,2,3,4,5,6]. The efficacy of antibiotics is superior to that of other drugs; thus, antibiotics are widely used [7]. However, the antibiotic resistance is currently a health emergency, so drug-resistant bacterial infections are becoming more common with a consequent increase in public spending [8,9]. About 60–70% of the existing antibiotics are not active against intracellular infections due to their low intracellular retention as a result of their poor permeability. Nanomaterials represent an attractive solution for the hydrophilicity barrier, because they can habitually penetrate cells, and increase their intracellular activity [10,11]. Small-size nanoparticles have the advantage of being characterized by a larger contact surface, which is able to enhance their penetration and therapeutic effects [12,13,14,15]. 

Among others nanomaterials, a good candidate is represented by silver nanoparticles (AgNPs) that can be easy functionalized [16,17], inducing hydrophilic behavior [18,19], antibacterial properties [13,20], and reduced inflammatory response [21], with low resistance phenomena in Gram-positive bacteria and Gram-negative bacteria [22,23,24]. The dimension and shape of AgNPs have a strong influence on antibacterial activity. In fact, a higher surface/volume ratio produces the higher rate of silver ions release [25]. AgNPs’ effects have been studied against the multidrug-resistant bacteria such as *P. aeruginosa, E. coli, Streptococcus pyogenes, S. aureus, Klebsiella pneumoniae, Salmonella* species, and *Enterococcus species* [26,27]. In these papers, the bactericidal action is mainly due to the inhibition of cell wall synthesis, nucleic acid synthesis, and protein synthesis mediated by the 30S ribosomal subunit. The strong bactericidal effect of AgNPs against the multidrug-resistant bacteria is mostly due to their multiple mechanisms to disrupt microbial cells [28]. Moreover, AgNPs can improve the antibiotic effects against *S. aureus* and *E. coli* [29]. 

Today, in medicinal practice, there are wound dressings, contraceptive devices, surgical instruments, bone prostheses, and dental implants that are coated or embedded with nanosilver [30,31,32,33]. Moreover, in the last decade, the research field of AgNPs has moved to the possibility of their use as an anticancer drug, due to their inherent cytotoxic effect on cancer cells [34].

However, the instability of silver nanoparticles limits their industrial application in several cases, and most of the methods to prepare AgNPs cause environmental pollution and low production efficiency. To overcome this problem, silver nanoparticles are usually loaded onto carriers [35]. 

Moreover, despite AgNPs having multiple mechanisms for antibacterial effects, recent studies showed bacterial resistance to them: the resistance evolves without any genetic changes; only phenotypic change is needed to reduce the nanoparticles’ colloidal stability and thus eliminate their antibacterial activity [36,37]. 

As a response to this problem, hybrids/composites with AgNPs dispersed on carriers or supports have been studied to enhance antibacterial activity compared with sole AgNPs: it is of significance to seek the optimal choice of carriers to combine with AgNPs in order to construct ideal antibacterial agents. Various AgNPs-based nanocomposites with different structures and morphologies have been developed up to now, such as an amorphous silica matrix dispersed with AgNPs [38], AgNPs core@silica shell [39], mesoporous silicas loaded with AgNPs [40], hollow mesoporous silica spheres with AgNPs in the cavity [41,42], fibers coated with AgNPs [43], etc. Although extensive efforts have been devoted to fabricating a lot of these AgNPs-based nanocomposites involving different carriers’ structures, there are still few systematic investigations on the effects of structures on antibacterial performance [44]. The use of drug delivery systems (DDS) has been proposed to overcome important issues in the release of active pharmaceutical molecules, such as unfavorable pharmacokinetics and biodistribution with a consequent decrease of side effects. 

Nanocarriers represent an innovative approach to overcome these issues [45,46]. Among other nanocarriers, such as liposomes, polymersomes, micelles, and polymer-based vesicles, the niosomes systems, non-ionic surfactant vesicles [46,47], have attracted attention from researchers because of their ability to encapsulate different kinds of drugs for the purpose of increasing their stability and efficacy. In fact, niosomes enable modulating the drug concentration loading in a range of interest for the biological applications (0.3–5.0 µg/mL for AgNPs) and to consent to drug-release control [48].

In this research study, AgNPs were synthetized using 3-mercapto-1-sodium propanesulfonate (3MPS) to induce hydrophilic behavior, improving the niosomal entrapment efficiency and reducing the bilayer destabilization. AgNPs were loaded in two different niosomes, Tween 20 and Span 20 ones, producing two different systems, namely NioTw20 + AgNPs and NioSp20 + AgNPs. A deep physical chemical characterization was carried out to obtain information on hydrophilic AgNPs and their influence on the preparation and characterization of Nio + AgNPs. Moreover, stability studies were performed in water, bovine serum, and human serum to assess their use in biological compartments. Hydrophilic and lipophilic probe release profiles were obtained in HEPES and in human serum. Both systems proved to be able to entrap AgNPs, are stable, and maintain the ability to entrap also hydrophilic or lipophilic model molecules, and so are promising systems for biotechnological applications.

## 2. Experimental

### 2.1. Materials and Methods

Silver nitrate (AgNO_3_, 99.5%, Sigma-Aldrich, St. Louis, MO, USA) and sodium borohydride (NaBH4, 98%, Sigma-Aldrich, St. Louis, MO, USA) were used for the synthesis of the nanoparticles. 3-mercapto-1propanesulfonic acid sodium salt (C_3_H_7_S_2_O_3_Na, 3MPS, 98%, Sigma Aldrich, St. Louis, MO, USA) was used as a capping agent. For all of the solutions, we used deionized water (electrical conductivity less than 1 µΩ/cm at room temperature) obtained from a Millipore Milli-Q water purification system. HEPES salt (sodium 2-(4-(2-hydroxyethyl) piperazin-1-yl) ethanesulfonate), cholesterol, Sephadex G75, Pyrene, DPH (1,6-diphenyl-1,3,5-exatriene), Span 20 (sorbitan monolaurate), Tween 20 (polyoxyethylene sorbitan monolaurate), human/bovine serum, calcein, and Nile Red were purchased from Sigma-Aldrich (Milan, Italy). All the other products and reagents were of analytical grade. All of the reagents were purchased from Sigma Aldrich and were used without further purification.

### 2.2. Preparation of AgNPs Loaded Niosomes

The AgNPs–3MPS synthesis (see Figure S1) consisted in a wet reduction of silver nitrate to metallic silver by means of sodium borohydride in the presence of 3MPS [17,18]. Briefly, a solution of AgNO_3_ in deionized water (0.200 g in 10 mL) was added in a flask with 3MPS water solution (0.830 g in 10 mL), and the mixture was maintained under stirring in argon atmosphere at room temperature for 10 min. Then, an NaBH_4_ water solution (0.220 g in 10 mL) was added dropwise, under vigorous stirring. The reaction mixture was allowed to react for 2 h. The obtained black product was centrifuged with deionized water three times (20 min, 5000 rpm), and the solid obtained was characterized. 

Several niosomal formulations by Tween 20 (NioTw20) or Span 20 (NioSp20) were prepared using AgNPs-3 MPS at different concentrations. Only the results obtained by selected samples in terms of the size, ζ-potential, entrapment efficiency, and stability features were reported and discussed. 

AgNPs–3MPS were loaded into Span 20 and Tween 20 niosomes through a protocol already used to internalize chemicals [49]. Niosomes were prepared using the thin film hydration method [50]. Span 20 or Tween 20 (15 mM) and cholesterol (15 mM) were dissolved in organic solvent mixture (chloroform/methanol 3:1 *v*/*v*). The solvent was evaporated using a rotary evaporator (VV2000, Heidolph, Schwabach, Germany) to form a thin “film”. The film was hydrated using 5 mL of AgNPs solution, which was then vortexed and sonicated at 60 °C and 18%/16% amplitude for 5 min using an ultrasonic microprobe (Vibra-Cell VCX-400, Sonics & Materials, Newtown, CT, USA). The unilamellar vesicular suspension was purified by gel filtration chromatography using Sephadex G75 (glass column of 50 × 1.2 cm) with HEPES buffer as the eluent. The purified vesicles were filtrated by using cellulose filters (pore size 1.2 μm).

### 2.3. Characterizations

The AgNPs water suspension has been characterized by means of UV-Vis collected using a Perkin-Elmer Lambda 19 and a Cary 100 spectrophotometer using quartz cuvettes. The dynamic light scattering (DLS) measurements on the AgNPs colloidal suspensions (0.200 mg/mL) at T = 25.0 ± 0.2 °C were performed by the Malvern Zetasizer Nano ZS90 instrument (Malvern, UK), as reported in previous studies [49,51]. The ζ-potential was calculated from the measured electrophoretic mobility by means of the Smolukovsky equation [52]. UV-Vis: λ_max_ (nm) 415; DLS: <2R_H_> 5 ± 2 nm; ζ-potential: −35 ± 2 mV.

The mean size, size distribution, and ζ-potential of empty and AgNPs-loaded niosomes were characterized by using DLS. UV-Visible spectroscopy was employed to evaluate the amount of AgNPs entrapped in niosomal formulations. The AgNPs’ entrapment efficiency was expressed as encapsulation yield, i.e., the percentage of nanoparticles loaded with respect to the total amount added for the preparation. Bilayer characterization has been carried out on empty Span 20 or Tween 20 niosomes and on AgNPs-loaded ones, employing DPH and pyrene (lipophilic probes) that provided different bilayer information (fluidity, microviscosity, and polarity). 

Span 20/Tween 20 (15 mM), cholesterol (15 mM), and DPH solution (2 × 10^−4^ M) were codissolved in chloroform/methanol, which was removed using a rotatory evaporator (VV2000, Heidolph, Schwabach, Germany), and then hydrated with HEPES buffer or AgNPs solution (0.5 mg/mL), with the same preparation methods as those mentioned above. A cellulose filter with a 450-nm cut-off (Spectrum, New Jersey, New Brunswick USA) was used to purify the DPH–niosomal formulations. Fluorescent measurements were performed (λ = 350–425 nm) using a luminescence spectrometer (LS5013, PerkinElmer) in order to obtain fluorescence anisotropy. The florescence anisotropy (r) was determined by using Equation (1) [53,54,55].

(1) $$A=\frac{I_{vv}-I_{vh}\times G}{I_{vv}+2I_{vh}\times G}$$

where *Ivv, Ivh, Ihv*, and *Ihh* are fluorescent intensities, and subscript *v* (vertical) and *h* (horizontal) represent the orientation of polarized light. The G factor is ratio of the sensitivity of the detection system for vertically and horizontally polarized light.

Pyrene-loaded niosomes were prepared by adding the probe (4 mM) to niosomes (Nio) components in order to obtain empty and Nio-AgNPs following the same preparation method as above. Pyrene is a florescence probe, whose monomer exhibited a spectrum characterized with five emission peaks (from I1 to I5) and excimer has only one peak (IE). The monomer and the excimer have different fluorescence signals, and the ratio between the several fluorescence intensities is directly related to the probe distribution in the bilayer. In particular, the ratio I1/I3, corresponding to the first and third vibration bands in the pyrene spectrum, is related to the polarity of the probe environment. Pyrene can form an intramolecular excimer based on the viscosity of the probe microenvironment [56], and it is estimated with the ratio IE/I3, where IE is the excimer intensity. The fluorescence signals emitted by pyrene-loaded niosome suspension were scanned (λ = 350–550 nm) using a luminescence spectrometer (LS5013, PerkinElmer) and the intensities of the excimer florescence (IE), first (I1), and third (I3) peak were recorded [57]. 

An atomic force microscopy (AFM) study of the morphology of the niosomes has been performed using a standard AFM setup (Dimension Icon, Bruker Inc., Milan, Italy) equipped with Si cantilevers suitable for tapping mode imaging. Samples have been prepared by depositing a droplet of solution on a clean monocrystalline Si surface and waiting until partial dehydration. AFM imaging has been performed in air and at room conditions.

Small-angle X-ray scattering (SAXS) experiments were performed at a ID02 high-brilliance beamline at the ESRF (Grenoble, France). The measured SAXS profiles report the scattered radiation intensity as a function of the momentum transfer, q = (4π/λ) sin (θ/2), where θ is the scattering angle and λ is the X-ray wavelength (0.1 nm). Analysis was carried out to obtain information on the dimension, homogeneity, and shape of the particles in solution. The form factors of niosomes have been reconstructed as unilamellar or multilamellar closed bilayers. Details are reported in the Supporting Information.

Biological studies were also carried out in the presence of bovine or human serum to evaluate the in vitro stability. NioTw20/AgNPs and NioSp20/AgNPs, as well as empty niosomes, were diluted in fetal bovine or human serum to obtain a final serum concentration of 45% at 37 °C. The average size, polydispersity index, and ζ-potential were evaluated by means of DLS at different time points (15 min, 30 min, 60 min, and 180 min) [58]. 

Stability studies up to 90 days at different temperatures—room temperature (RT) and 4 °C—were carried out by DLS to asses the dimension and ζ-potential of empty niosomes and Nio-AgNPs [58].

Release studies were carried out following the release calcein from empty niosomes and Nio-AgNPs using a Fluorimetric apparatus.

In order to obtain information about the ability of Nio to release lipophilic and hydrophilic probes, also in presence of AgNPs, Nile Red (lipophilic fluorescent probe) and calcein (hydrophilic fluorescent probe) release studies were carried out.

The hydrophilic probe (calcein 10^−2^ M) was added to the film during hydration, and the excess of calcein was purified by gel filtration chromatography, as already mentioned [58].

On the contrary, due to its apolar nature, the fluorescent probe Nile Red was added to the vesicle components at a final concentration of 10^−3^ M, and it will be located in the hydrophobic bilayer.

The in vitro release experiments were carried out using dialysis tubes (molecular weight cut-off 8000 and 5.5 cm^2^ diffusing area) at 37 °C in HEPES buffer (10 mM, pH 7.4). The Nile Red/calcein concentration was measured using the UV spectrophotometer/luminescence spectrometer at different time points over 1–24 h. 

In vitro release experiments were performed at 37 °C, and the defined volume of vesicle dispersions was included in dialysis sacs (cut-off 8.000) with a fixed diffusing area (5.5 cm^2^) adding to a niosomal formulation of 45% human serum or HEPES (in order to maintain the same probe concentration). The probe concentration was detected in the outer solution at fixed time intervals (0 h, 1 h, 2 h, 3 h, 4 h, 5 h, 6 h, 7 h, 8 h, and 24 h) by means of the Fluorometric apparatus, taking into account the dilution factor. 

## 3. Results and Discussion

**Figure 1:**
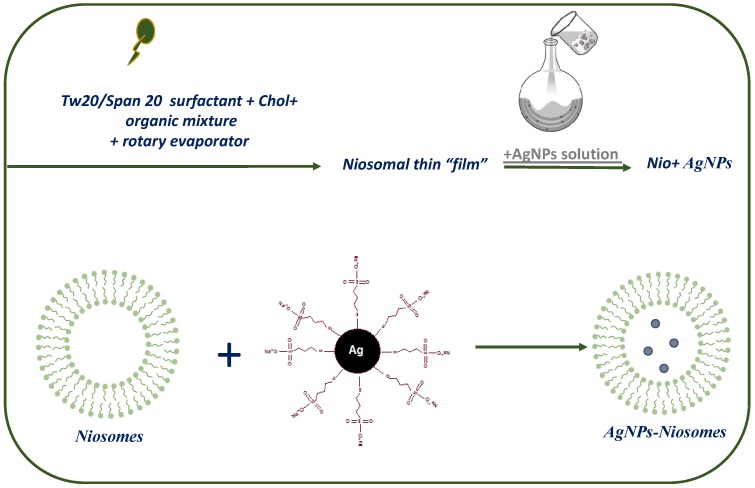
Preparation of niosomes hydrated with silver nanoparticles (Nio-AgNPs).

AgNPs were functionalized by 3MPS to induce high hydrophilicity and to control the shape and dimension in the range of 2 to 5 nm by means of UV-Vis and DLS measurements, as already reported [17,18]. In fact, the hydrophilicity is a crucial feature to obtain the insertion in the aqueous core of the niosomes. Even the small dimensions are a key parameter to ensure the inclusion and above all the stability of the final hybrid system, as also reported in the literature [48]. The AgNPs were loaded into niosomes, as schematized in Figure 1.

According to characterization results obtained, the best samples selected were Tween 20/Span 20 niosomes hydrated with AgNPs at the 0.5 mg/mL concentration.

The first comparison between empty niosomes and Nio-AgNPs was done by analyzing their hydrodynamic diameter, ζ-potential, and PDI (polydispersity index) by DLS. The results are shown in Table 1. In both niosomal formulations, with Tween 20 or Span 20, all the parameters analyzed by DLS are preserved after the addition of AgNPs. The empty samples based on Tween 20 and Span 20 show differences in dimensions, due to the different internal structures determined by the different surfactants employed for niosomal preparation. In particular, Span 20 niosomes, as expected [59], are bigger than Tween 20 ones, and show more negative ζ-potential.

The entrapment efficiency of AgNPs in vesicular systems was evaluated by means of the calibration curve (see Figure S2), and the results are reported in Table 2.

The data obtained indicate that the entrapment efficiency for the two systems is not the same; Span 20 niosomes are more efficient than Tw20 niosomes, which is probably related to their different internal structures and/or capacity (aqueous volume available to host AgNPs). Moreover, these values allow assembling systems with a silver concentration in the range of interest for biological applications (0.3–5.0 µg/mL), as reported in literature [48]. The results obtained by bilayer characterization studies, such as fluidity, microviscosity, and polarity (Table 3), show no variation in the evaluated bilayer properties, thus demonstrating that no interactions occur between the niosomal double layer and AgNPs, which probably will be located inside aqueous compartments.

**Figure 2:**
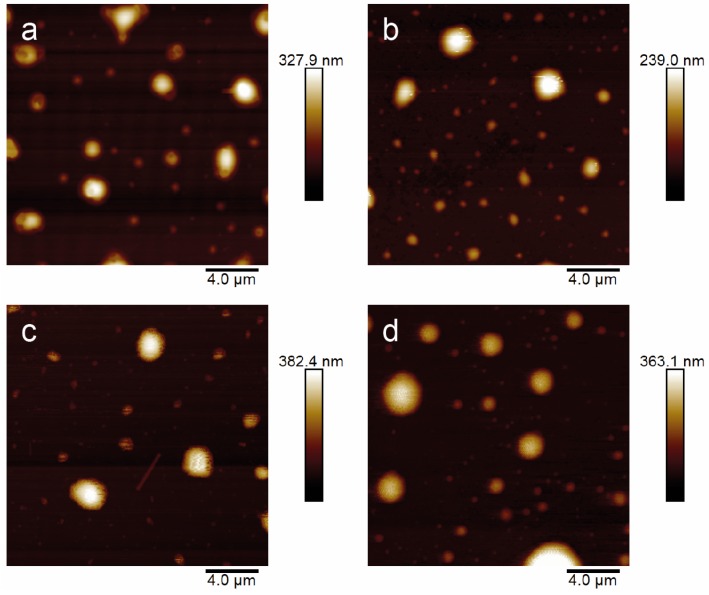
Atomic force microscopy (AFM) images related to: NioTw20 (**a**), NioTw20 + AgNPs (**b**), NioSp20 (**c**), and NioSp20 + AgNPs (**d**).

Morphological studies were performed on empty niosomes and Nio-AgNPs. Representative AFM images of the samples are shown in Figure 2. The morphological characterization indicates that niosomes have regular spherical shapes. Probably due to the intrinsic limitations related to sample preparation, the size of niosomes seems highly dispersed, according to PDI values by DLS analyses, the bigger particles being probably the results of the agglomeration of individual niosomes.

In the deposited sample, large amorphous particles are visible, likely resulting from the coalescence of several vesicles on the substrate surface as well as the possible partial dehydration making the vesicles lose their original spherical shape. By a visual inspection, Tween-based niosomes, i.e., NioTw20 (Figure 2a) and NioTw20 + AgNPs (Figure 2b), are smaller than the Span-based ones, i.e., NioSp20 (Figure 2c) and NioSp20 + AgNPs (Figure 2d), which is in qualitative agreement with the measured hydrodynamic diameters reported in Table 1. Conversely, the effect of the presence of AgNPs on the size cannot be appreciated in both the Tween and Span-based formulations, considering the relatively small variations evaluated by DLS and the dispersion of the size in the AFM samples. 

In order to characterize the local structure of niosomes, SAXS measurements were performed on Tween 20 and Span 20 niosomes that were empty and loaded with AgNPs.

**Figure 3:**
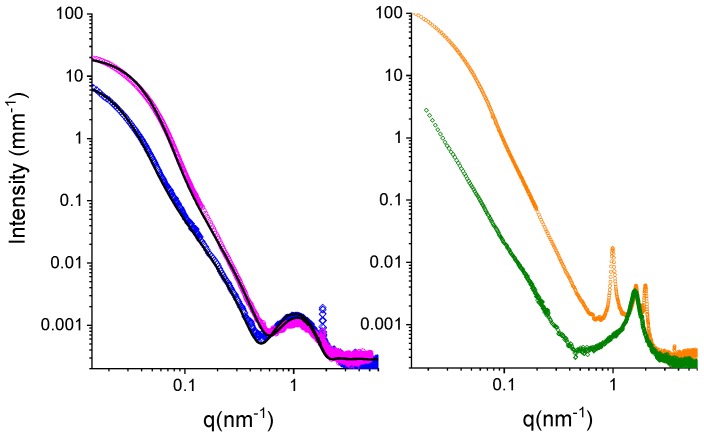
SAXS spectra of Tween 20 and Span 20-based niosomes. Panel A. Tween 20-based empty niosomes (blue diamonds) and Nio-AgNPs (magenta dots). Fitting curves have been obtained by modeling the particle as an internal solvent core surrounded by a surfactant closed bilayer (for details, see Supporting Information). Panel B. Span 20-based empty niosomes (green diamonds) and Nio-AgNPs (orange dots).

In Figure 3, the intensity spectra are reported for Tween 20-based nanoparticles (panel A) and for Span 20-based nanoparticles (panel B) in a wide q range (0.014 nm^−1^ ≤ q ≤ 6 nm^−1^), corresponding to distances from 150 nm to the nm. Differences in the intensity profiles are clearly visible for the two systems in all the regions of the spectra. Besides the feature of the form factor of the particles in solution, a small diffraction peak at 1.84 nm^−1^, corresponding to a characteristic distance of 3.41 nm, is well known, and stems from the presence of cholesterol crystallites in surfactants/cholesterol mixtures [60]. Crystallites could be either excluded from the bilayers, in peripheral contact, for example, or included in the bilayers, as segregated structures.

Tween 20-based particles display a niosomes shape that is characterized by a local bilayer structure with a thickness of about 6 nm, being the hydrophobic core of 2.5 nm and the two hydrophilic layers of 1.6 nm and of 2 nm, respectively. Moreover, the absence of peaks due to multilamellar organization reveals that the adopted structure is unilamellar. 

In the presence of AgNPs, Tween 20 niosomes keep a local unilamellar structure, with unaffected structural features. On the other hand, in the low-q region of the spectrum, the scattered intensity increases by one order of magnitude. The form factor of the unilamellar closed particles can be modeled, replacing the internal water with a higher electron density solvent, confirming the presence of AgNPs entrapped inside the aqueous core of the niosomes.

Span 20-based aggregates display the characteristic features of closed lamellar-type particles. Nevertheless, the local structure of Span 20-based niosomes is quite different from the Tween 20-based one. A broad intensity peak is clearly visible at q = 1.6 nm^−1^, together with a very broad left shoulder. The position of the peak corresponds to a characteristic distance of d = 3.9 nm, which is compatible with twice the length of a Span 20 (sorbitan monolaurate) molecule. The results indicate that Span 20 niosomes are multilamellar closed particles, with a water core surrounded by a peculiar layered shell: several adjacent concentric bilayers are in close contact, heads to head, without any water penetration. The scattered intensity profile of Span 20-based loaded niosomes, as reported in Figure 3 (panel B), presents a pronounced increase in the low-q region, which is a sign of the presence of AgNps enclosed in the internal aqueous core of the niosomes. The increase is definitely higher than the one observed in Tween 20-based Nio-AgNPs, suggesting that Span 20 is more efficient in entrapping metallic NPs. On the local scale, two additional peaks at q = 1 nm^−1^ and q = 2 nm^−1^ are visible, revealing a swollen multilamellar structure with a characteristic distance of d = 6 nm, coexisting with the tight one at d = 3.9 nm. The 6-nm distance is typical for lipid multilamellar structures and is also found in Tween^®^20-derivatives/cholesterol niosomes [60]. The presence of AgNPs induces the partial disjunction of adjacent bilayers with increased water penetration.

**Figure 4:**
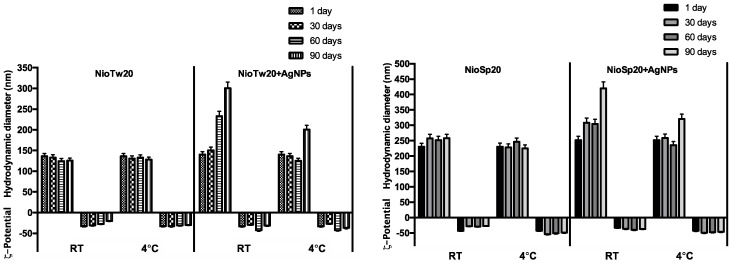
Stability studies of empty niosomes and Nio-AgNPs at room temperature and 4 °C.

Stability studies of NioTw20/NioTw20 + AgNPs and NioSp20/NioSp20 + AgNPs were performed to compare their behavior at different temperatures, as shown in Figure 4. Empty niosomes are stable both at room temperature and at 4 °C, while Nio-AgNPs showed a different behavior in terms of the dimensional increase at RT conditions, which was not confirmed at a 4 °C storage temperature. The colloidal stability at 4 °C is higher because of the reduced collision events of the dispersed particles, and hence coalescent phenomena.

**Figure 5:**
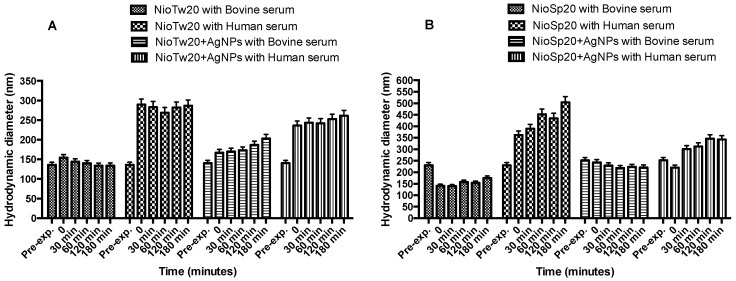
Niosomal biological stability at 37 °C in different media. (**A**) NioTw20/AgNPs in bovine and human serum; (**B**) NioSp20/AgNPs in bovine and human serum.

The effect of the serum (human and bovine) is another important element to evaluate in order to define the interaction between vesicles and biological fluids. Experiments were performed at 37 °C evaluating the size and ζ-potential (data not shown) variations by DLS analysis up to 3 h (Figure 5). During the time interval analyzed, the same trend is observed for all the vesicles. Vesicles in 45% bovine serum do not show a dimensional increase, while in 45% human serum, the trend is different. The different protein composition of human serum is the reason for the attractive interaction between a negatively charged niosomal surface and proteins. These interactions are strong enough to observe the same populations over the three hours. This result, which has to be investigated further, is consistent with the fact that niosomal vesicles should act as an anchor for the blood proteins. Indeed, after incubation with human serum, plateau values of about 280 nm and 380 nm are reached. This time evolution suggests that the human serum composition is responsible for a faster kinetic toward the equilibrium rather than the bovine one, because of a different protein pattern [57,61].

In order to confirm the ability of Nio to release lipophilic and hydrophilic probes, also in the presence of AgNPs, calcein and Nile Red release studies were carried out. 

**Figure 6:**
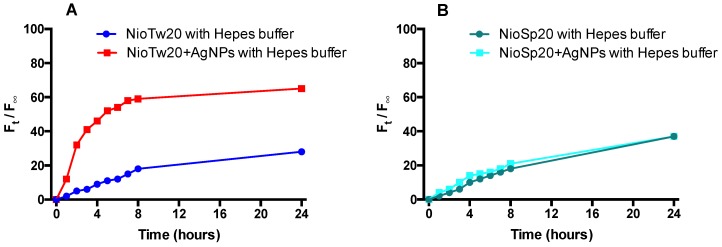
Calcein release studies in HEPES buffer at 37 °C from: (**A**) NioTw20/AgNPs; (**B**) NioSp20/AgNPs.

**Figure 7:**
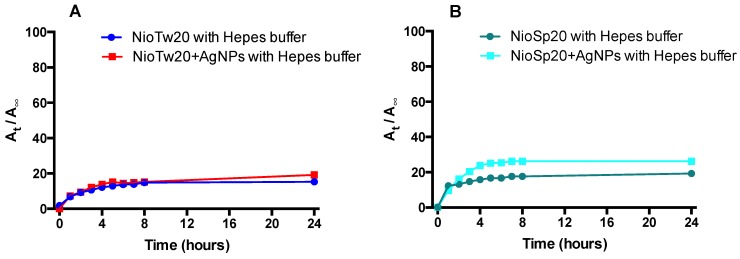
Nile Red release studies in HEPES buffer at 37 °C (**A**) NioTw20/AgNPs; (**B**) NioSp20/AgNPs.

Figure 6 and Figure 7 show the release profiles calcein and Nile Red. In each experiment, the calcein release data from empty niosomes and NioTw20 AgNPs were compared in order to evaluate the influence of the silver nanoparticles, which were entrapped in the same compartment, on the hydrophilic probe release. The calcein entrapment in both samples was comparable. Experiments were carried out at 37 °C both in HEPES and human serum. The results obtained in human serum are not reported because the calcein release was not significant (20%), which was probably due to the coating and masking effects of the serum, which make it difficult to quantify the calcein release in the external medium. In HEPES buffer, the presence of AgNPs influences the release profile of calcein only in the NioTw20 formulation. As demonstrated by SAXS analyses, only a double layer is present, so it is more susceptible to silver nanoparticles’ destabilization. Calcein release by this sample is around 65% in 24 h, with respect to 30% by NioSp20/AgNPs, where the presence of different bilayers can make calcein release more difficult.

On the contrary, in both samples, the Nile Red release profiles that were obtained in HEPES at 37 °C are comparable, because of the lipophilic nature of the probe, as shown in Figure 7.

The number of bilayers to cross is the limiting step for hydrophilic probe release, while it is not the limiting step for the lipophilic probe that is located in the bilayer, and will be barely released for the poor affinity with the aqueous external medium.

The morphological structure of the two different niosomal formulations and the presence or not of AgNPs, could influence the release profiles of the two probes. 

## 4. Conclusions

In this study, hydrophilic AgNPs were loaded in two different niosomes, producing two systems, namely NioTw20 + AgNPs and NioSp20 + AgNPs. A deep physical–chemical characterization was carried out to obtain information on the influence of AgNPs on the preparation and features of niosomal formulations. First of all, the DLS studies confirm the nanosize and stability of both systems in water. Moreover, the entrapment efficiency for the two systems was investigated, and it was more efficient for Span 20 than Tw20 niosomes, which was probably related to their different internal structures. Microviscosity and polarity investigations demonstrated that no interactions occurred between the niosomal double layer and the AgNPs, which were probably located inside aqueous compartments. The SAXS data confirmed the presence of the AgNPs located inside the aqueous compartment of the two niosomal systems, and also allowed highlighting the different structures of their double layers. The morphological characterization indicates that the niosomes maintained spherical shapes. Moreover, stability was confirmed in water, bovine serum, and human serum. Moreover, hydrophilic and lipophilic probe release profiles were obtained in HEPES and in human serum. In conclusion, both systems evidenced the entrapment of AgNPs: NioTw20-AgNPs and NioSp20-AgNPs. The two systems are stable in water, bovine serum, and human serum, and maintain the ability to entrap also hydrophilic or lipophilic model molecules. This work demonstrates that the niosomes’ features are not altered by AgNPs loading, and confirms that these niosomal formulations are good candidates for the delivery of AgNPs together with other drugs, opening new promising ways for their biotechnological applications.

## Figures and Tables

**Table 1:** Hydrodynamic diameter, ζ-potential, and polydispersity index (PDI) of different niosomal formulations. NioTw20: niosomal formulations by Tween 20, NioSp20: niosomal formulations by Span 20.

Samples ID	Hydrodynamic Diameter (nm) ± SD	ζ-Potential (mV) ± SD	PDI ± SD
NioTw20	136.1 ± 2.0	−32.8 ± 0.3	0.38 ± 0.01
NioTw20 + AgNPs	140.3 ± 3.9	−33.1 ± 1.4	0.40 ± 0.01
NioSp20	230.2 ± 5.9	−42.7 ± 2.3	0.35 ± 0.01
NioSp20 + AgNPs	251.7 ± 6.0	−42.9 ± 1.2	0.40 ± 0.01

**Table 2:** The entrapment efficiency in percentage of AgNPs in niosomes.

Samples ID	Entrapment Efficiency (%)
NioTw20 + AgNPs	<1
NioSp20 + AgNPs	4

**Table 3:** Bilayer characterization results of niosomes (Nio) and Nio-AgNPs.

Samples ID	Fluidity (Anisotropy)	Microviscosity (I_E_/I_3_)	Polarity (I_1_/I_3_)
NioTw20	0.10	0.90	0.90
NioTw20 + AgNPs	0.11	0.90	0.90
NioSp20	0.10	1.01	0.94
NioSp20 + AgNPs	0.11	1.03	0.90

## Supplementary Materials

The following are available online at https://www.mdpi.com/2079-4991/9/8/1177/s1, Figure S1: (**a**) Synthetic scheme for AgNPs-3MPS; (**b**) Uv-vis spectrum of AgNPs-3MPS in water with SPR at λ = 425 nm; (**c**) <2R_H_> = 8 ± 3 nm of AgNPs-3MPS in water; (**d**) ζ-potential = −35 ± 2 mV of AgNPs-3MPS in water; Figure S2: Calibration curve of AgNPs; SI Details about Small angle X-ray Scattering (SAXS).
