# Supplementary material for: Hydrophilic Silver Nanoparticles Loaded into Niosomes: Physical–Chemical Characterization in View of Biological Applications
Source: Nanomaterials (Basel). 2019 Aug 17;9(8):1177. doi: 10.3390/nano9081177 (PMC6724070; doi:10.3390/nano9081177)
Supplement: Supplementary file 1 [file nanomaterials-09-01177-s001.pdf]

SUPPORTING MATERIALS

Hydrophilic Silver Nanoparticles Loaded into Niosomes: Physical–Chemical Characterization in View of Biological Applications

Federica Rinaldi [1], Elena del Favero [2], Johannes Moeller [3,†], Patrizia Nadia Hanieh [4], Daniele Passeri [5], Marco Rossi [5], Livia Angeloni [5], Iole Venditti [6,*], Carlotta Marianecci [4,*], Maria Carafa [4,‡] and Ilaria Fratoddi [7,‡]

1 Center for Life Nano Science@Sapienza, Istituto Italiano di Tecnologia (ITT), 00161 Rome, Italy
2 Department of Medical Biotechnology and Translational Medicine, University of Milan, 20122 Milano, Italy
3 ESRF Grenoble Fr, 38043 Grenoble, France
4 Department of Drug Chemistry and Technologies, Sapienza University, 00185 Rome, Italy
5 Department of Basic and Applied Sciences for Engineering, Sapienza University, 00185 Rome, Italy
6 Department of Sciences, Roma Tre University, 00146 Rome Italy
7 Department of Chemistry, Sapienza University, 00185 Rome, Italy
* Correspondence: iole.venditti@uniroma3.it (I.V.); carlotta.marianecci@uniroma1.it (C.M.); Tel.: +39-06-57333388 (I.V.); Tel.: +39-06-49913970 (C.M.)
† Current affiliation: European XFEL, Holzkoppel 4, 22869 Schenefeld, Germany.
‡ Co-last authors.

a)

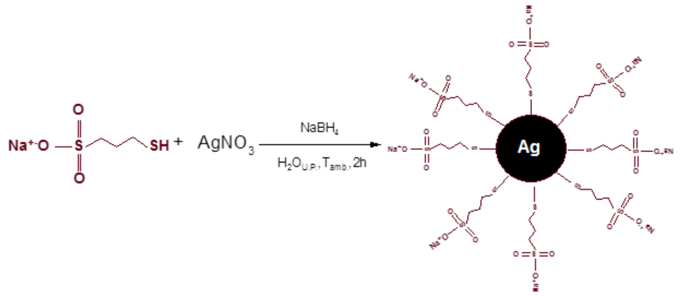

b)

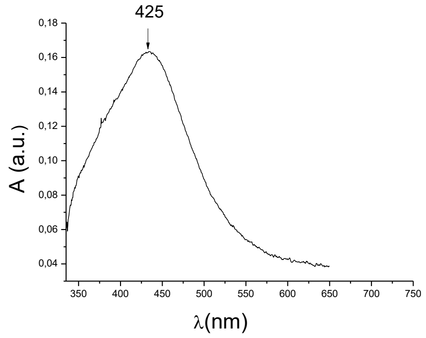

c)

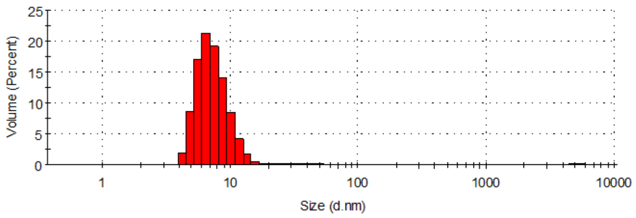

d)

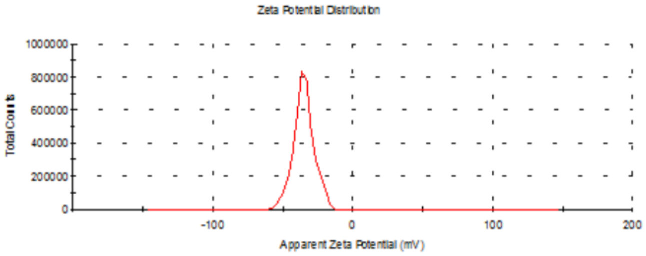

Figure S1: a) Synthetic scheme for AgNPs-3MPS; b) Uv-vis spectrum of AgNPs-3MPS in water with SPR at λ = 425 nm; c) <2RH>= 8 ± 3 nm of AgNPs-3MPS in water; d) ζ-potential = -35 ± 2 mV of AgNPs-3MPS in water.

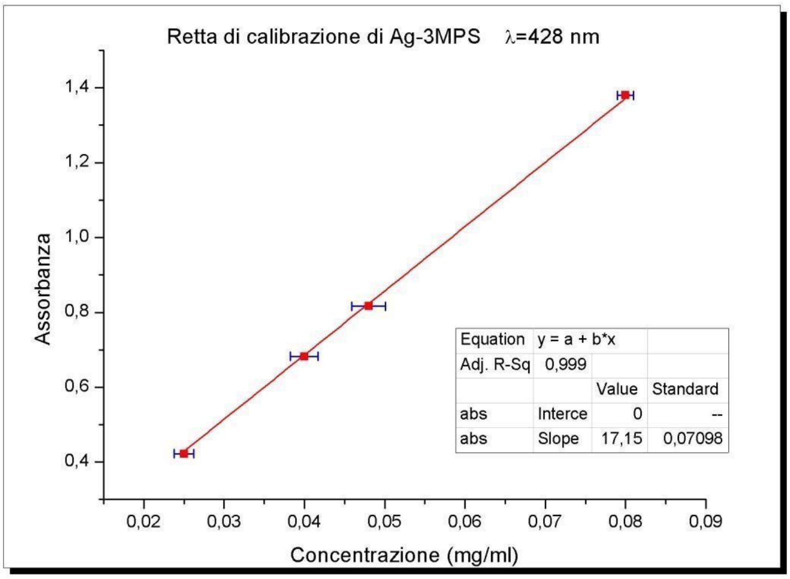

Figure S2: Small angle X-ray Scattering (SAXS).

SI Details about Small angle X-ray Scattering (SAXS)

SAXS experiments were performed at ID02 high-brilliance beamline at the ESRF (Grenoble, France) with a beam wavelength 0.1 nm, in the region of momentum transfer, $q = (4\pi/\lambda)\sin(\theta/2)$, $0.014\ nm^{-1} \leqslant q \leqslant 6\ nm^{-1}$, where θ is the scattering angle. Samples were put in plastic capillaries (KI-beam, ENKI, Concesio, Italy) mounted horizontally onto a six-places sample holder, allowing for nearly contemporary measurements on sample and reference cells in the same environmental conditions. All measurements were performed at 25 °C. The exposure time of each measurement was very short, 0.1 s, in order to minimize any eventual radiation damage. Anyway, several frames were collected on each sample, with 1 s sleeping time, carefully compared and mediated if superimposable within experimental error. The measured SAXS profiles report the scattered radiation intensity as a function of the momentum transfer, q. Several spectra relative to the empty cells and the solvent were taken, carefully compared and subtracted to each sample spectrum. To investigate a wide q region, SAXS spectra relative to different q ranges were compared and joined (sample-detector distances 1m and 6m). Analysis was carried out to obtain information on dimension, homogeneity, shape of the particles in solution. The form factors of Tween 20 niosomes have been modelled by the form factor of polydisperse core-multishell particles:

$$P(q) \propto \left[\sum_i 3V_i(\rho_i - \rho_{i-1})\frac{\sin(qR_i) - qr_i\cos(qR_i)}{(qR_i)^3}\right]^2$$

V_i indicates the volumes of each concentric sphere with radius R_i and scattering electron density ρ_i. Spectra have been reconstructed with SasView application (M. Doucet et al. SasView Version 4.2, Zenodo, 10.5281/zenodo.1412041) as an internal solvent core surrounded by three-shell closed layers, the external hydrophilic headgroups region (thickness = 2 nm), the hydrophobic chain region (thickness = 2.5 nm) and the internal hydrophilic headgroups region (thickness = 1.6 nm). Polidispersity was modelled with a Schulz distribution (0.4) for the radius and with a Gaussian distribution (0.1) for the bilayer chain region. For AgNPs loaded niosomes the intensity profile has been reconstructed with the same structural parameters except for the internal water core (ρ = 334 e/nm^3) that was replaced with a solvent with higher electron density (ρ = 345 e/nm^3). In the case of Span 20 AgNPs loaded niosomes the increase in intensity is greater and compatible with an internal core characterized by an electron density ρ = 380 e/nm^3, thus revealing the presence of a larger amount of entrapped AgNPs.
